# Tree Shrews as an Animal Model for Studying Perceptual Decision-Making Reveal a Critical Role of Stimulus-Independent Processes in Guiding Behavior

**DOI:** 10.1523/ENEURO.0419-22.2022

**Published:** 2022-11-24

**Authors:** Chuiwen Li, Kara M. McHaney, Per B. Sederberg, Jianhua Cang

**Affiliations:** 1 Department of Psychology, University of Virginia, Charlottesville, VA 22904; 2 Department of Biology, University of Virginia, Charlottesville, VA 22904; 3 Department of Biology and Department of Psychology, University of Virginia, Charlottesville, VA 22904

**Keywords:** sequential sampling model, decision-making, tree shrew, timed racing diffusion model

## Abstract

Decision-making is an essential cognitive process by which we interact with the external world. However, attempts to understand the neural mechanisms of decision-making are limited by the current available animal models and the technologies that can be applied to them. Here, we build on the renewed interest in using tree shrews (*Tupaia belangeri*) in vision research and provide strong support for them as a model for studying visual perceptual decision-making. Tree shrews learned very quickly to perform a two-alternative forced choice contrast discrimination task, and they exhibited differences in response time distributions depending on the reward and punishment structure of the task. Specifically, they made occasional fast guesses when incorrect responses are punished by a constant increase in the interval between trials. This behavior was suppressed when faster incorrect responses were discouraged by longer intertrial intervals. By fitting the behavioral data with two variants of racing diffusion decision models, we found that the between-trial delay affected decision-making by modulating the drift rate of a time accumulator. Our results thus provide support for the existence of an internal process that is independent of the evidence accumulation in decision-making and lay a foundation for future mechanistic studies of perceptual decision-making using tree shrews.

## Significance Statement

Despite decades of work in the field of decision-making, we still have no clear brain-wide model of how perceptual decisions are formed and executed. A major reason for this lack of understanding is the limited animal models in decision-making studies. Here, we have successfully established a rigorous perceptual decision-making paradigm in tree shrews, and evaluated their choice and response-time behaviors with both summary statistics and trial-level computational modeling. Our results suggest that an endogenously-driven decision process, in addition to standard stimulus-dependent evidence accumulation, is necessary for interpreting the observed behavior. Our study thus underscores the importance of characterizing additional factors that affect decisions and encourages future investigations using tree shrews to reveal the neural mechanisms underlying these cognitive processes.

## Introduction

Decision-making is a vital cognitive process, playing an important role in many brain functions such as categorization, learning, memory, and reasoning. Among different forms of decision-making, perceptual decision-making, where decisions are based on sensory stimuli, is a simple yet informative task that is particularly amenable to experimental studies. Visual stimuli are often used because the visual system is arguably the best studied sensory system, thus advantageous for understanding perceptual decision-making from sensation to action.

Considering decision-making is a dynamic process with complex combinations of distinct underlying variables, researchers have frequently applied sequential sampling models (SSMs) to interpret and decompose decision behaviors. These models assume that the evidence (i.e., a variable depending on the sensory stimulus strength) is accumulated through time, and a corresponding choice is made when the accumulated evidence passes a threshold. By defining these stochastic accumulation processes, SSMs can simulate decisions and response times (RTs) with the stimulus as the input. The discovery of “ramping neurons” during decisions in many brain regions provides neural evidence for these models (Horwitz and Newsome, 1999; Roitman and Shadlen, 2002; Ding and Gold, 2010; Mante et al., 2013). Despite the models’ effectiveness in a wide range of applications, variants of the SSM make different predictions regarding what decision variables (bias, threshold, time perception, etc.) are involved and how they interact with each other (Ratcliff, 1978; Usher and McClelland, 2001; Brown and Heathcote, 2005; Cisek et al., 2009). More importantly, the neural mechanisms of these variables and their interactions remain largely unknown, which typically require studies in animal models.

**Figure 1.**
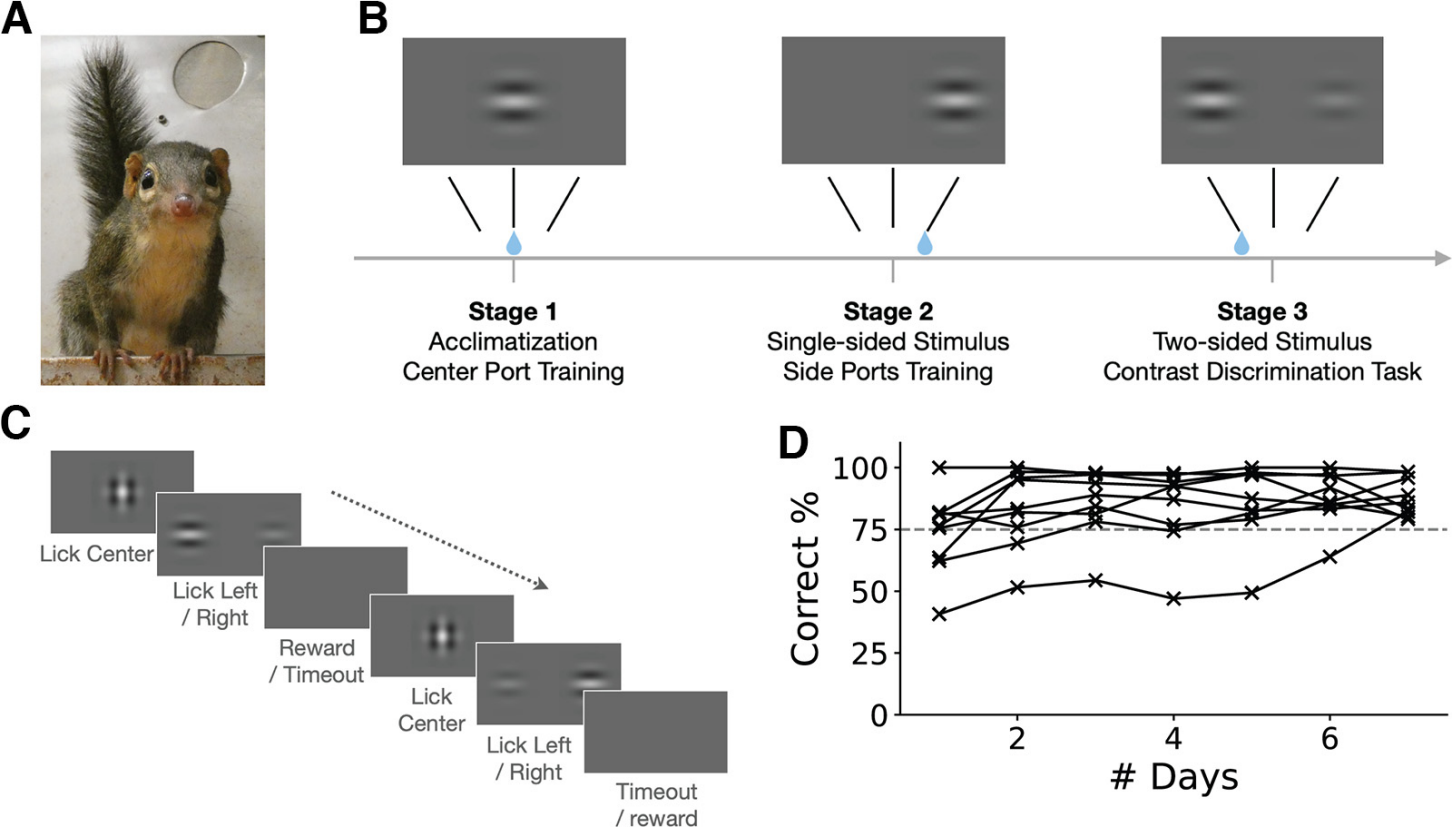
Experimental design. ***A***, A photograph of a tree shrew in the home cage. ***B***, A schematic of the training procedure. ***C***, The contrast discrimination task. The animal needs to choose the side that has a higher contrast gabor and report the choice by licking the corresponding port. ***D***, Learning curve of individual animals. The *y*-axis is the response accuracy for the easiest condition on each day. Day 1 refers to the first day of training with two-sided gabor stimulus. Dashed gray line, 75% accuracy. Most animals reached this level by day 2 and all by day 7.

Monkeys and rodents (mostly rats and mice) are commonly used in decision-making studies, with respective advantages and drawbacks. Monkeys are closely related to humans, but they are expensive and limited in availability, thus difficult to study or control individual differences. Furthermore, most modern “circuit-busting” opto-genetic and chemo-genetic techniques are not yet routinely used in primates. On the other hand, recent use of rodents, especially mice, has significantly advanced our understanding of decision-making (Odoemene et al., 2018; International Brain Laboratory et al., 2021; Ashwood et al., 2022). However, mice and rats are nocturnal animals with poor eyesight, making them less than ideal for visual tasks. In addition, rodents often learn visual tasks slowly (Aoki et al., 2017; Urai et al., 2021), costing both time and effort to obtain high quality data. Here, we use a different animal model, tree shrews (*Tupaia belangeri*; Fig. 1*A*) for visual decision studies. Under the order of *Scandentia*, tree shrews are evolutionarily closer to primates than rodents are (Yao, 2017). They are diurnal, have an excellent acuity, and display visual system complexity similar to primates (Petry and Bickford, 2019). Earlier studies have shown that they could be reliably trained to perform visual (color, orientation, spatial frequency, temporal frequency, etc.) discrimination tasks (Casagrande and Diamond, 1974; Petry et al., 1984; Petry and Kelly, 1991; Callahan and Petry, 2000; Mustafar et al., 2018). In addition, tree shrews are of lower cost, smaller, and have a faster reproduction cycle than monkeys, making them more accessible. Finally, modern viral, genetic, and imaging techniques are being applied in tree shrews with a better success than in primates (Lee et al., 2016; Li et al., 2017; Sedigh-Sarvestani et al., 2021; Savier et al., 2021). Taken together, tree shrews have the potential to advance the understanding of neural mechanisms underlying perceptual decision-making. In this study, we seek to establish a rigorous perceptual decision-making paradigm for tree shrews, and to characterize the decision-making features, including both response accuracy and response time, in this animal model quantitatively with both summary statistics and trial-level computational modeling.

## Materials and Methods

### Contrast discrimination task

We trained in total of nine (male = 7, female = 2) freely moving tree shrews to perform a two-alternative forced choice (2AFC) contrast discrimination task (Fig. 1*C*). At the beginning of each trial, a visual stimulus of two orthogonal overlapping α-transparent gabors appeared at the screen center to indicate that the tree shrew could lick the center port to initiate the trial. After initiation, the center stimulus disappeared, and two side gabor patches were presented immediately on the left and right of the screen. Tree shrews needed to choose the side with a higher contrast by licking the corresponding lick port. This self-initiation design helped to ensure that the animals were focused from the beginning of each trial and allowed us to record accurate RTs, which were calculated as the duration between the stimulus (two side gabors) appearance and the side-port lick detection. Once a choice lick was detected, the stimulus would disappear from the screen. We adopted a free-response structure that if no choice was detected, the stimulus would be on for an infinite amount of time.

Intertrial intervals (ITIs) were randomly drawn from a truncated normal distribution with a mean of 0.6, a SD of 1, a lower bound of 0.5, and an upper bound of 0.7 (unit: seconds). For correct responses, liquid reward (50% grape juice) was given right after the animals reported their choices. The reward volume was determined by the duration of the valve opening, which was randomly drawn from a truncated normal distribution with a mean of 0.1, a SD of 0.06, a lower bound of 0.2, and an upper bound of 0.4 (unit: seconds). The speed of liquid flow was 150 μl/s. The average reward volume in one correct trial was 33 μl (0.22 s). The random ITI and random reward duration helped the animals to stay engaged in the task.

For incorrect responses, two protocols were used to generate a delay as a punishment. (1) A fixed delay of 4 s was used in the first group of tree shrews for all incorrect responses. If the animal licked the center port during the delay (i.e., blank screen licks; detected in 0.8 s periods), a penalty of 0.8 s was then added to the delay, with a maximum of 8 s for the total delay. (2) An exponential decay function (Equation 1) was applied in the second group of animals to generate a between-trial delay based on the trial-level RT:

(1) $$T=\frac{1}{s}e^{-\frac{RT-l}{s}},$$

where *T* is the between-trial delay, *RT* is the response time of the current incorrect trial, and *l* and *s* are the location and scale parameters, which shift and scale the function in the stimulus generation code. For all animals, we used *l *=* *0.1, *s *=* *1.7. For the blank screen lick penalty, 1.5 s was added for every center-port-lick, with the total delay being *Max*(*T*, *t_passed_* + *penalty*), and no upper limit. To determine the potential effect of these two delay paradigms, we calculated the reward rate using the data of a representative animal from the first group of tree shrews (Eq. 2): the response accuracy of each RT bin was fitted with a sigmoid function, which was then used to calculate the theoretical reward per unit time (pulse/s).

(2) $$RR(t)=\frac{Acc(t)}{Acc(t)\times t+(1-Acc(t))\times (t+Delay(t))},$$

where *RR*(*t*) is the reward rate for a response time of *t*, *Acc*(*t*) is the response accuracy (i.e., ratio of correct choices) under this response time *t* obtained from the observed data, *Delay*(*t*) is the intertrial delay for incorrect responses, which is four for the fixed-delay rule or follows the exponential decay function defined above (Eq. 1) for the exponential-delay rule.

### Animal training and data collection

Tree shrews were first acclimated to the behavior box for 1–2 d. For most animals (seven out of nine), water restriction started at this stage of training (stage 1). For the other two animals, water restriction started a couple of days before acclimation. Two approaches of water restriction were used: (1) we gradually reduced their water intake from baseline (20–40 ml/d) to 5–10 ml/d by limiting the availability of drinking water; (2) we used citric acid (CA; Urai et al., 2021) water in their home cage to reduce water intake and gradually increased its concentration from 2% to 4%. The progress of water restriction depended on the animals’ weight loss, water-intake baseline, and tolerance, to make sure that they were motivated to stay focused on the task for at least 25 min/d, and at the same time, not experiencing any health issue (weight ≥90% × baseline). Depending on the animals’ acclimation and learning speed, the water restriction progress (2–7 d) could extend to stage 2 and even 3 before reaching a stable restriction level.

During stage 1, a single gabor stimulus would be shown right above the center lick port. After the gabor appeared, the animals could lick the center port at any time to trigger a liquid reward (grape juice diluted with water in a 1:1 ratio). Each tree shrew was left in the behavior box to learn to use the center port for no more than 20 min every day for acclimation, but this stage usually took only 1 d (20–40 trials per day). Having learnt to get liquid reward from the center port, the animals progressed to the next stage. At stage 2, the contrast discrimination task was set up with contrast pairs of 1.0 (full contrast) versus 0.0 (zero contrast), i.e., a single side stimulus was shown. The goal of stage 2 was to train the animals to use the left and right lick ports. Liquid reward from the center port was gradually reduced to zero within ∼50 trials. Animals usually perform 100–300 trials per day at this stage. Once they learned and had a stable correct rate of >75%, they progressed to stage 3. Note that most animals learned very fast and graduated both stages 1 and 2 within 2 d.

At stage 3, we first gave the animals an easy condition by using contrast pairs of 1.0 versus 0.1, and gradually mixed in other pairs of smaller contrast differences, finally achieving the stimulus set we use in the formal data collection. During this stage of training, we also adjusted the ratio of easy (e.g., comparing the highest and lowest contrast) and difficult (same or similar contrast) trials for each animal. By including sufficient easy trials and limiting the number of equal-contrast trials, we were able to keep the animals motivated to keep doing the task. For equal contrast trials, the correct answer was randomly assigned to left or right, so that the animals still had 50% chance to get a reward in these trials. At this stage, the animals performed 500–600 trials per day. Some animals could finish it within 30 min, while some of the others needed as long as 1 h, especially when they produced large numbers of incorrect choices (giving rise to more penalty time) or they started to lose patience and focus (giving rise to more idling time). To control the frustration level, we would stop the training when the duration was over 1 h. At this time, some animals (50%) also developed biased behavior by making most choices to the same side. We discouraged this behavior by automatically adjusting the probability of left/right trials depending on their real-time performance. For example, we calculated the proportion of choosing rightward in the previous 10 trials, denoted as *Pr*. The probability of the next trial being rightward was 1 – *Pr*. This real-time bias correction quickly discouraged the biased behavior in the tree shrews.

After the animals achieved a stable (three to five consecutive days) overall accuracy ≥60% (at this time, the accuracy is expected to be lower because of the existence of equal contrast trials and other difficult trials), we collected data for consecutive days (500–600 trials per day) to reach at least 100 repeats for each condition of contrast discrimination. The data were first culled by applying a three SD outlier removal on the Box–Cox transformed response time distribution in preprocessing. The remaining trials were used in further analysis.

All animal procedures were performed in accordance with the University of Virginia animal care committee’s regulations.

### Stimulus and apparatus

The experiment program was written in Python and the stimuli were generated and presented with the State Machine Interface Library for Experiments (SMILE; https://github.com/compmem/smile). The Gabor patch size was 28∘, and the spatial frequency was 0.2 cpd. The stimulus screen had a 1280 × 1024 resolution and 60-Hz refresh rate, and was γ-corrected. It was set at a distance of 15 cm from the animal. There were six levels of stimulus contrasts ranging from 0.08 to 0.99, which were evenly-spaced. All combinations of left and right contrasts are presented in a randomized order.

The lick-detector circuit (adapted from Marbach and Zador, 2017), and reward-valve control circuit (adapted from https://bc-robotics.com/tutorials/controlling-a-solenoid-valve-with-arduino/) were controlled with an NI USB-6001 multifunction I/O device (https://www.ni.com/en-us/support/model.usb-6001.html). The Plexiglas behavior box was L: 40 cm × W: 22 cm × H: 20 cm with a transparent window on the front side to allow the animals to watch the screen.

### Data analysis and models

To test the relationship between RT and contrast difference, we fitted a mixed effect linear regression model with RT as the dependent variable, the absolute contrast difference between left and right stimuli as the independent variable, and individual animal as the group variable, using the statsmodels library in Python.

We fitted the behavioral data with two sequential sampling decision-making models, the timed racing diffusion model (TRDM) and the racing diffusion model (RDM), and compared their performance using a Bayesian approach. TRDM contains three independent accumulation processes, namely two evidence accumulators and one time accumulator (or “timer”), whereas RDM only has the two evidence accumulators. The probability density function (PDF) [*f*(*t*)] and cumulative distribution function [*F*(*t*)] for each evidence or time accumulation process are defined by the inverse Gaussian (Wald) distribution in Equation 3:

(3) $$f(t|\rho ,\sigma ,\alpha ,t_{0})=\frac{\alpha }{\sigma \sqrt{2\pi {\left(t-t_{0}\right)}^{3}}}exp\left(-\frac{{\left[\alpha -\rho (t-t_{0})\right]}^{2}}{2\sigma^{2}(t-t_{0})}\right)F(t|\rho ,\sigma ,\alpha ,t_{0})=\Phi \left(\frac{\rho (t-t_{0})-\alpha }{\sigma \sqrt{t-t_{0}}}\right)+exp\left(\frac{2\alpha \rho }{\sigma^{2}}\right)\cdot \Phi \left(-\frac{\rho (t-t_{0})+\alpha }{\sigma \sqrt{t-t_{0}}}\right),$$

where *t* is the response time, *ρ* is the mean drift rate, *σ* is the within-trial variability of the drift rate, *α* is the threshold (which was fixed to 1.0), *t*_0_ is the nondecision time, Φ is the cumulative distribution function of a standard normal distribution (Heathcote, 2004; Hawkins and Heathcote, 2021).

The mean drift rate (*ρ*) of each evidence accumulator was calculated using the following equation (Eq. 4), taking into consideration both the stimulus difference and their total strength:

(4) $$\begin{array}{l} \rho_{l}=v_{0}+v_{d}(s_{l}-s_{r})+v_{s}(s_{l}+s_{r})\\ \rho_{r}=v_{0}+v_{d}(s_{r}-s_{l})+v_{s}(s_{l}+s_{r}), \end{array}$$

where *ρ_l_* and *ρ_r_* are the mean drift rate of the left and right evidence accumulators, *v*_0_ is the baseline drift rate, *s_l_* and *s_r_* are the contrasts of left and right stimuli, *v_d_* is the coefficient of the contrast difference term, *v_s_* is the coefficient of the contrast summation term (van Ravenzwaaij et al., 2020).

The accumulators race against each other. If one of the evidence accumulators reaches the threshold first, a corresponding choice is made. If the time accumulator reaches the threshold first, one of the options will be chosen randomly with a partial dependence on which evidence is greater at that time point. This is done through a process controlled by a parameter *γ*, ranging from 0 to 1, with 1 being fully dependent on the evidence accumulated up until that point, and 0 being completely random regardless of the accumulated evidence. Other parameters of the model include *ρ_t_*, *ω*, and *t*_0_, as described in Table 1.

**Table 1:** Priors of free parameters in tested models

Parameter	Description	Prior
*ω*	Bias	*IL*(0, 1.4)
*t*_0,_ *_c_*	Nondecision time of choice	*IL*(0, 1.4)
$v_{0},v_{s},v_{d}$	Drift rate coefficients of choice	*LN*(1.56, 1.5)
$\rho_{t}^{*}$	Mean drift rate of timer	*LN*(1.56, 1.5)
$\eta_{c},\eta_{t}^{*}$	Within-trial variability	*LN*(1.56, 1.5)
*γ* ^*^	Mixture between random and evidence-based timer-induced decision	*IL*(–1, 1.0)

*^IL^* inverse logit distribution.

*^LN^* log normal distribution.

* parameters only exist in TRDM.

The best fitting parameters of the two models for each animal are shown in Extended Data Tables 1-2 and 1-3. We also tested the relationship between RT and contrast difference using nonmodel statistics described in Extended Data Table 1-1.

10.1523/ENEURO.0419-22.2022.tab1-1 Extended Data Table 1-1 Statistical table. Download Table 1-1, DOC file.

10.1523/ENEURO.0419-22.2022.tab1-2 Extended Data Table 1-2 TRDM best fitting parameters of each animal. Download Table 1-2, DOC file.

10.1523/ENEURO.0419-22.2022.tab1-3 Extended Data Table 1-3 RDM best fitting parameters of each animal. Download Table 1-3, DOC file.

To apply Bayesian inference, we first defined the “priors,” the belief of the true parameter values before data observation, by assigning a probability distribution for each of the parameters based on previous experience (Table 1; Kirkpatrick et al., 2021). We then used the observed data to update the prior distributions, to achieve a more constrained posterior distribution of what parameters could have generated the observed data for each model. Posterior samples were generated with the differential evolution Markov chain Monte Carlo (DE-MCMC; Ter Braak, 2006; Turner and Sederberg, 2012; Turner et al., 2013) algorithm, which was shown to be computationally efficient. This was implemented by the RunDEMC library (https://github.com/compmem/RunDEMC). We set 10*k* (*k* is the number of parameters) parallel chains for 200 iterations in the burn-in stage and 500 iterations to sample the posterior.

Specifically, we apply a standard Metropolis–Hastings algorithm to accept or reject proposed samples from the posterior. Here, a new parameter proposal is evaluated by comparing its posterior probability with that of the current proposal, with the probability of accepting a new proposal:

(5) $$P(accept)=\frac{P(D|\theta^{\prime })P(\theta^{\prime })}{P(D|\theta )P(\theta )},$$

where *D* represents the observed data, *θ′* is the new proposal, *θ* is the current proposal, *P*(*D*|θ′) and P(D|θ) are the likelihoods calculated with Equation 6, and *P*(*θ′*) and *P*(*θ*) are the priors.

To calculate the likelihood P(D|θ) of observing the data *D* given the parameters *θ*, we multiply the likelihoods of observing each choice and RT as determined by the model probability density function (PDF) defined by the parameters *θ*. For example, the PDF for observing a *left* response with a decision time *t* is defined by the following equation (Heathcote, 2004; Hawkins and Heathcote, 2021):

(6) $$PDF_{left}(t)=f_{E,left}(t)\left(1-F_{E,right}(t)\right)\left(1-F_{T}(t)\right)+P_{T}f_{T}(t)\left(1-F_{E,left}(t)\right)\left(1-F_{E,right}(t)\right)P_{T}=\gamma F_{X}(0)+\frac{1}{2}\left(1-\gamma \right)X∼N\left(\rho_{r}t-\rho_{l}t,\sqrt{2{\left(\eta_{c}\sqrt{t}\right)}^{2}}\right),$$

where *f*(*t*) and *F*(*t*) are the density and distribution functions defined above, *f_E_* and *F_E_* are for the evidence accumulators, while *f_T_* and *F_T_* are for the time accumulator. *F_X_* is the cumulative distribution function for the random variable *X*, and *X* follows a normal distribution defined by the difference in evidence accumulator distributions. *ρ_l_* and *ρ_r_* are the mean drift rate for left and right evidence accumulators, *η_c_* is the within-trial variability of the drift rate for the evidence accumulators.

Finally, to compare the performance of the two models, we first calculated the Bayesian information criterion (BIC) values (Eq. 7) of each model fitting result:

(7) $$BIC=k\text{ln}(n)-2\text{ln}\left(L(\hat{\theta })\right),$$

where *k* is the number of parameters, *n* is the number of data points, $L(\hat{\theta })$ is the maximum likelihood of the model’s fit to the data. Then we approximated the Bayes factor (BF) with BIC as in Equation 8 (Kass and Raftery, 1995):

(8) $$BF_{ij}\approx \text{exp}\left(-\frac{1}{2}(BIC_{i}-BIC_{j})\right),$$

where *BIC_i_* and *BIC_j_* are BIC values for Model i (in this case the TRDM) and Model j (the RDM) respectively. *BF_i_*_,_*_j_* > 1 means evidence is in favor of Model i over Model j. *BF_i_*_,_*_j_* > 3, 20, 150, correspondingly ln (*BF_i_*_,_*_j_*) > 1, 3, 5, indicates positive, strong, very strong evidence for Model i over Model j, respectively (Lodewyckx et al., 2011).

### Code accessibility

Python code for preprocessing and running TRDM/RDM models are included in the Extended Data 1.

10.1523/ENEURO.0419-22.2022.ed1 Extended Data 1 Code for analysis and modeling. fit_rdm.py fit_trdm.py single_animal_preprocessing.ipynb waldrace.py Download Extended Data 1, ZIP file.

## Results

### Tree shrews quickly learned to perform a contrast discrimination 2AFC task

We trained a total of nine (male = 7, female = 2) tree shrews to perform a 2AFC contrast discrimination task (Fig. 1). The 2AFC design was chosen over other classic paradigms such as “Go/no-Go” tasks because it eliminates the asymmetry between responses for different options. Also, we designed the trials to be self-initiated and self-paced by the animals, to obtain precise response time (RT) data for comprehensive behavioral analysis. During training, freely moving tree shrews were first acclimated in the behavioral box with a single gabor stimulus appearing at the center or either side of the screen (Fig. 1*B*). After the animals learned the association between the stimulus and liquid reward, often within 1–2 d, two gabors of different contrasts were introduced with the higher contrast one indicating the location of the reward (Fig. 1*C*). All the tree shrews were able to learn the task and reach an accuracy >75% for the easiest condition within one week (Fig. 1*D*). In fact, most of them reached 75% accuracy within 2 d. It is worth noting that, once the animals reached a good performance, the overall difficulty was increased progressively. In other words, the “easiest” condition often became more difficult in successive days. However, the animals’ performance was stably above 75%, indicating that they had learned the rule of the task, instead of the specific stimuli, within a very short period. These observations thus highlight the impressive learning capability of tree shrews and indicate that they can be a promising animal model in cognitive neuroscience research.

### Tree shrews showed different behaviors under two training schemes

**Figure 2.**
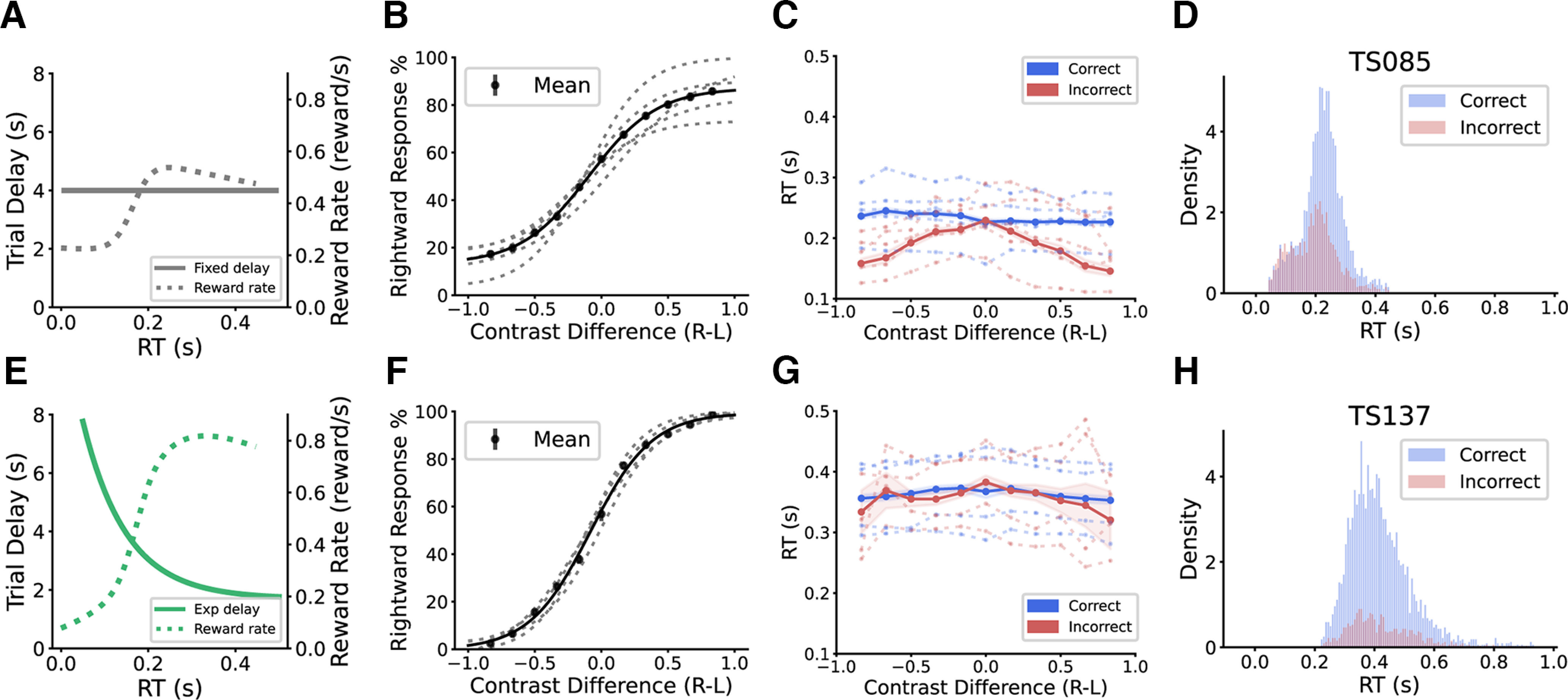
Tree shrews show different behaviors under two training schemes. ***A***, A fixed delay of 4 s (solid line) was used in training one group of animals. The dashed line shows the theoretical reward rate under this fixed delay. ***B***, Psychometric curve of animals from this training scheme. Contrast difference: right contrast (*R*) – left contrast (*L*). Gray dashed line, Individual animals. Black solid line, Average across animals. ***C***, Response time (RT) as a function of contrast difference. Dashed line, Individual animals. Solid line, Average across animals. The shaded area is 95% confidence interval. ***D***, RT density histogram from a representative animal. Correct and incorrect trials are separately plotted. ***E***, An exponential decay delay scheme (solid line) was applied in another group. The dashed line shows the theoretical reward rate under this scheme. ***F–H***, Same as ***C–E*** but for the second group. Figures 2-1 and 2-2 show the RT distributions of individual animals from the fixed-delay group and exponential-delay group respectively.

In the first group of animals (*n* = 5; male = 4, female = 1), a fixed trial delay of 4 s was used to punish incorrect responses (Fig. 2*A*). All animals were able to learn the task. An increase in difficulty (i.e., a decrease of contrast difference between the two stimuli) induced an expected drop of response accuracy (Fig. 2*B*). However, task difficulty did not have a significant effect on the response time (RT) in correct trials (mixed effect linear regression, *β* = 0.008*^a^*, *p *=* *0.125; Extended Data Table 1-1), whereas the RT in incorrect trials increased with task difficulty (Fig. 2*C*, mixed effect linear regression, *β* = –0.075*^b^*, *p *<* *0.001). This result is different from previously reported RT trend in humans, monkeys, and mice (Roitman and Shadlen, 2002; Palmer et al., 2005; Philiastides et al., 2011; Dmochowski and Norcia, 2015; Jun et al., 2021; Orsolic et al., 2021), where increasing task difficulty usually resulted in an increase in RT in correct trials. We examined the RT distribution of individual animals and saw a bimodal-like shape in most animals (*n* = 4 out of 5) in this group (Fig. 2*D*; Extended Data Fig. 2-1), instead of the more common log-normal distribution (Ratcliff, 1978; Smith and Ratcliff, 2004). Furthermore, the first small peak of the RT distribution contained a similar proportion of correct and incorrect trials, while the second peak had many more correct than incorrect trials. This bimodal distribution suggested two possible modes in the behavioral responses, a “fast-guessing” mode of random performance and a slower mode where an animal was more “engaged” in the task.

10.1523/ENEURO.0419-22.2022.f2-1 Extended Data Figure 2-1 Response time distributions of the individual animals from the fixed-delay group. Download Figure 2-1, TIF file.

To discourage the animals from “fast guessing,” we employed an exponential decay trial delay for incorrect responses in the second group (*n* = 4; male = 3, female = 1; Fig. 2*E*). The exponential decay delay would punish fast incorrect responses more than slow incorrect ones, at a more aggressive level than the fixed trial delay procedure (Fig. 2*A*,*E*). All animals in this group were again able to learn the task quickly (Fig. 2*F*,*G*). Notably, the overall RT was substantially slower compared with the fixed-delay group, indicating the effectiveness of the new trial delay paradigm. Furthermore, the RTs in correct trials showed a slightly increasing trend with task difficulty (mixed effect linear regression, *β* = –0.021*^c^*, *p *=* *0.001), while the effect on the incorrect RT became less prominent than for the fixed-delay group (mixed effect linear regression, *β* = –0.046*^d^*, *p *=* *0.014). When examining the RT distribution of individual animals, we saw one-peak log-normal distributions, similar to what was reported in other species, and a clear above-chance accuracy across the entire range (Fig. 2*H*; Extended Data Fig. 2-2). These behavioral data thus demonstrate that the tree shrews responded to the two trial delay schemes with different behaviors.

10.1523/ENEURO.0419-22.2022.f2-2 Extended Data Figure 2-2 Response time distributions of the individual animals from the exponential-delay group. Download Figure 2-2, TIF file.

### Non-evidence-accumulation mechanism is crucial to interpreting tree shrew behaviors

The above behavioral data suggest the involvement of a process in addition to evidence collection during decision-making. One possibility is a time accumulation process where the animals had an internal time threshold on the task, and they would rush into a more or less random choice if the time threshold was reached before accumulating enough evidence to guide the choice. This time limit would be different under the two trial delay paradigms: shorter under fixed delay, thus leading to many fast guesses. To test the plausibility of this explanation, we turned to cognitive models of decision-making.

**Figure 3.**
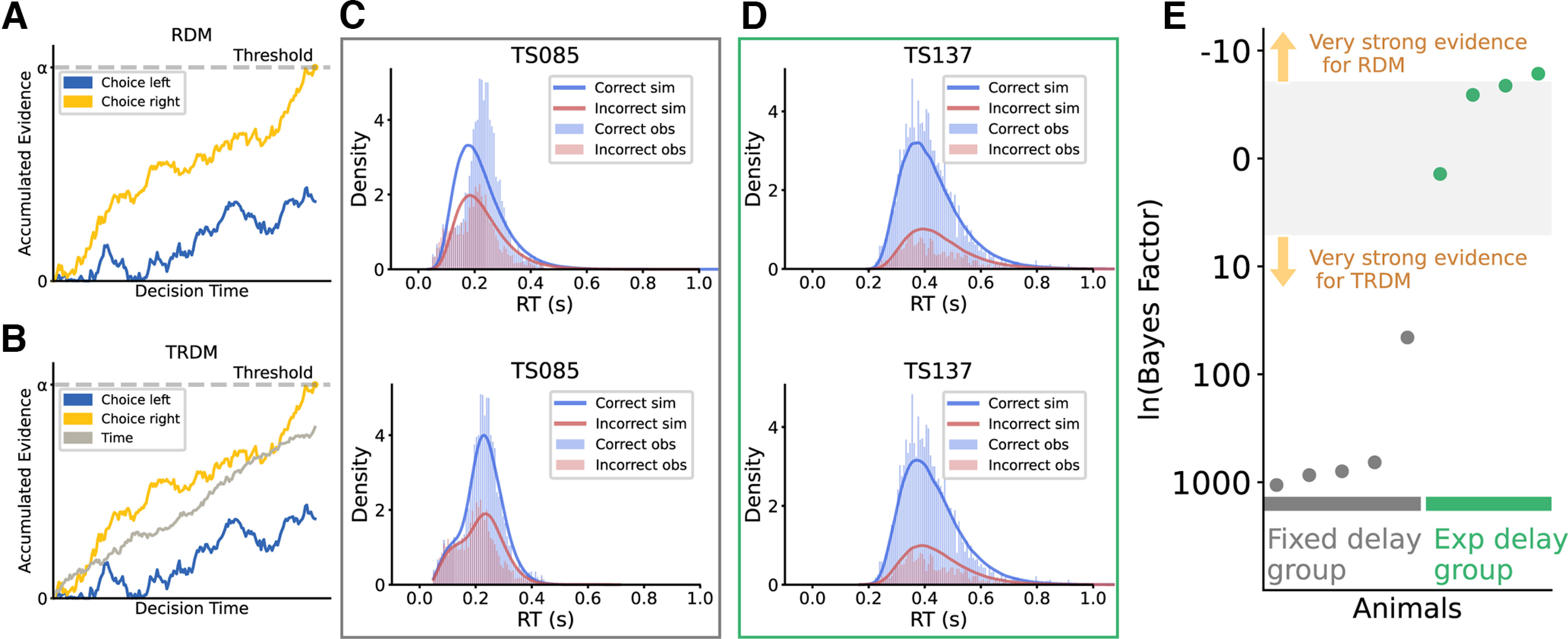
Modeling results suggest that evidence accumulation combined with a timing mechanism better fits tree shrew decision-making behavior. ***A***, ***B***, Racing diffusion model (RDM; ***A***) and timed racing diffusion model (TRDM; ***B***). Blue trace, The evidence accumulator for left choice. Yellow trace, The evidence accumulator for right choice. Gray trace, The time accumulator. The two evidence accumulation processes race against each other. In these schematics, the accumulator for right stimuli (yellow) reaches the threshold first, resulting in a rightward choice. ***C***, Observed (histograms) and simulated (lines) RT distribution for the representative animal from the fixed-delay group. Top, RDM simulation. Bottom, TRDM simulation. ***D***, Observed and simulated RT distribution for the representative animal from the exponential-delay group. Top, RDM simulation. Bottom, TRDM simulation. ***E***, Estimated log Bayes factor comparing the two models’ performance. Positive values favor TRDM, while negative values favor RDM. Gray dots represent the animals from the fixed-delay training, and green dots represent the exponential-delay group. The upper and lower edges of the gray shaded area represent the lower limit for “very strong” evidence [*ln*(*BF*)* *=* *5].

We fitted two models, racing diffusion model (RDM) and timed racing diffusion model (TRDM; Hawkins and Heathcote, 2021), to the data obtained from individual animals. In a 2AFC task, the RDM describes 2 independent evidence accumulators racing against each other. When one of the accumulators first reaches the threshold, a corresponding choice is made (Fig. 3*A*). The TRDM has one additional accumulator that tracks time (Fig. 3*B*). If the time accumulator reaches the threshold before the evidence accumulators, a decision is made based on the current accumulated evidence with a certain probability *γ*. We fixed all the accumulation thresholds to be 1. A fast time accumulator was thus effectively equal to a short time limit as described above. The two models allowed us to test whether an additional timing mechanism can better explain tree shrew decision behaviors.

We used a Bayesian approach for model fitting (Ter Braak, 2006; Turner and Sederberg, 2012; Turner et al., 2013), and then simulated choice and RT data with the best fitting parameters to visualize the goodness of fit. We found that the RDM captured the RT distribution of the exponential-delay group well, but failed to fit the fixed-delay group (Fig. 3*C*,*D*, top panels). On the other hand, the TRDM fitted well to both groups (Fig. 3*C*,*D*, bottom panels). To quantify their performance difference, we estimated the Bayes factor (BF) of the two models for each animal (Fig. 3*E*). For animals in the fixed-delay group, the values of ln(BF) were extremely high, ranging from 45 to 1062, providing overwhelming support for the TRDM. These values were much higher than 5, which is a conventional threshold for “very strong” evidence for one model over the other in Bayesian modeling (Lodewyckx et al., 2011). For the exponential-delay group, the evidence favored the RDM for three out of the four tree shrews, although the magnitude of evidence was not nearly as strong [*ln*(*BF*) ranging from −6 to 1]. It should be noted that Bayes factor in our estimation punishes complex models that have more parameters. As a result, despite the similar performance of the two models in fitting the exponential-delay group data, the RDM had the advantage of simplicity, thus leading to the winning BF.

**Figure 4.**
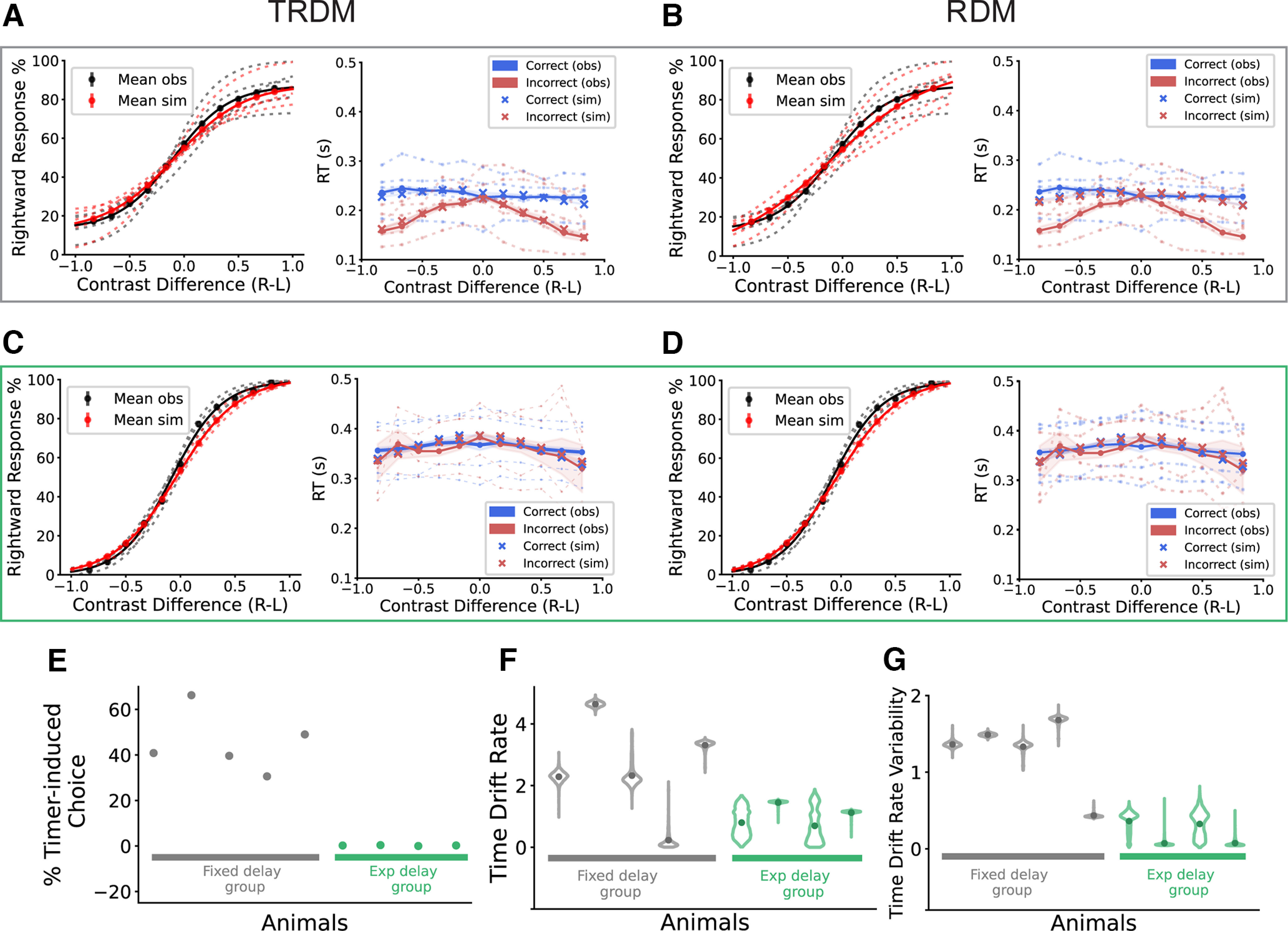
Model simulation of the psychometric curves and associated response time, and the posterior of the timer-related parameters. ***A***, TRDM simulation for the fixed-delay group. Left, Observed (black) and simulated (red) psychometric curves for individual animals (dotted lines) and the group average (solid lines). The simulations were done with the best fitting parameters of the TRDM. Right, Observed (dots, solid lines, and dotted lines) and simulated RT function (“x”). Dotted lines, Individual animals. Solid lines, Group average. ***B***, RDM simulation for the fixed-delay group. ***C***, TRDM simulation for the exponential-delay group. ***D***, RDM simulation for the exponential-delay group. ***E***, Percentage of timer-induced choice calculated from the TRDM-simulated data for each animal. ***F***, The posterior distribution of the time accumulator mean drift rate (*ρ_t_*) for individual animals from the TRDM fitting. The dot in each distribution indicates the mean value. ***G***, Same as ***F***, but for the drift rate variability of the time accumulator (*η_t_*). Figure 4-1 shows the decomposed simulation data of TRDM for one example animal.

We then simulated choice and RT data with the best fitting parameters (Extended Data Tables 1-2 and 1-3) for each animal using the winning model, to visually check the goodness of fit. Figure 4 illustrates that the TRDM fit the data of the fixed-delay group well (Fig. 4*A*), and the RDM was able to reproduce the behavior of the exponential-delay group (Fig. 4*D*), for both the psychometric curves and the RT-contrast relationship. Consistent with the result in Figure 3, the TRDM was also able to fit the psychometric curves and the RT-contrast relationship for the exponential-delay group (Fig. 4*C*), similarly to the RDM, while the RDM failed to capture the RT-contrast relationship for the fixed-delay group (Fig. 4*B*). The fact that the behavior of both groups could be explained by the TRDM supported the involvement of the non-evidence-accumulation process during tree shrew visual decision-making, and this process can be manipulated by applying different trial delay rules.

The models allowed us to track down the generating mechanism of the simulated data, i.e., whether each decision was initiated by an evidence accumulator or the timer crossing the threshold. We separated the TRDM-simulated data for each animal according to the generating mechanism, and found the timer and evidence accumulators contributed to two separate RT peaks. Extended Data Figure 4-1 shows the comparison between simulated data and observed data for an example tree shrew from the fixed-delay group (Fig. 2*D*). The results indicated that the fast RTs were largely generated by the timer (Extended Data Fig. 4-1*A*). In addition, when examining the simulated RTs for correct choices generated by evidence accumulators only, they increased with the task difficulty (Extended Data Fig. 4-1*D*), similar to what has been previously reported in humans, monkeys, and mice (Roitman and Shadlen, 2002; Palmer et al., 2005; Philiastides et al., 2011; Dmochowski and Norcia, 2015; Jun et al., 2021; Orsolic et al., 2021). These model results suggest that the tree shrews learned the visual decision-making task, and they had similar behaviors as other animals when “engaged” in the task. Moreover, the timer-driven random choices explained the plateau of a nonperfect accuracy, even in the easiest conditions (Extended Data Fig. 4-1*C*).

10.1523/ENEURO.0419-22.2022.f4-1 Extended Data Figure 4-1 Decomposition of an example animal’s simulated RT distribution by the TRDM. ***A***, The simulated RTs for one example animal (TS085) from the first group are divided into four groups: evidence accumulator generated RT for correct (blue) and incorrect (pink) responses, and time accumulator generated RT for correct (green) and incorrect (yellow) choices. Compared with the observed data (***B***), the plots show that the TRDM interprets the first peak (fast RT) in the RT distribution as generated by the time accumulator. ***C***, Simulated psychometric curves generated by the evidence accumulators and the time accumulator. ***D***, Evidence accumulator simulated RT as a function of contrast difference. Download Figure 4-1, TIF file.

Next, for each tree shrew, we quantified the percentage of timer-induced choices from the TRDM-simulated data (Fig. 4*E*). As expected from the above analysis, all of the animals from the fixed-delay group showed many timer-induced choices (ranging from 30% to 66%), while the value was near zero for every animal in the exponential-delay group. To understand what decision variables were altered by the change of delay rule, we examined the posterior distribution of the parameters in the TRDM. The posteriors of the timer-related parameters showed a general trend of higher mean drift rate for the time accumulator (*ρ_t_*) and higher time drift rate variability (*η_t_*) in the fixed-delay group than in the exponential-delay group (Fig. 4*F*,*G*). The two parameters work together to determine the accumulation speed of time during decision-making, with the fixed-delay group having faster timers. The model results therefore proposed a possible mechanism that the exponential delay worked by slowing down the time accumulation process in the tree shrews, which resulted in far fewer “timer-induced” fast responses with compromised accuracy, and more correct responses guided by the evidence accumulation process.

## Discussion

In this study, we aimed to and succeeded in establishing a response-time paradigm of perceptual decision-making for tree shrews. The behavioral results showed that tree shrews are able to perform a contrast-discrimination perceptual decision task and generate informative choice and response time data. Model-based analyses suggest that, other than the choice-related evidence accumulation process, additional mechanisms, presumably mechanisms that keep track of time, are involved in the decision-making process depending on the specific design of trial delay because of incorrect responses. This new animal model will facilitate future decision-making studies with fast learning, reliable behaviors, increased availability, and more modern techniques.

We carefully considered two points when designing the behavioral paradigm. First, we adopted a 2AFC framework, where two alternative options match symmetrically with two response targets. In other widely used tasks, there often exists asymmetry in either responses or stimulus categories, which can be problematic when interpreting different behaviors. For example, Go/no-Go tasks involve an action (“go”) and a suppression of action (“no-go”) as two responses, which are likely driven by different neural circuits. Such tasks have thus become more suitable for studying impulsion and inhibition (Eagle et al., 2008; Dong et al., 2010; na Ding et al., 2014). On the other hand, yes/no tasks offer two asymmetric stimulus categories as options, which are likely represented differently at the neural level (Wentura, 2000; Donner et al., 2009). In comparison, a multiple alternative forced choice framework is better in perceptual decision-making studies. Second, we designed the task to be self-initiated and self-paced by the animals. Self-initiation ensures that the animals are focused during the stimulus presentation, and self-pacing encourages them to respond without delay once they reach a decision. Compared with the commonly-used design where the stimuli show up automatically and animals can respond at any time point within a fixed response window, our design allowed us to collect precise response times in addition to choice data. Response times are particularly useful because they are continuous (whereas choice data are discrete) and are more informative when characterizing decision behaviors. For example, fast correct responses have potentially different mechanisms from slow correct responses, which would be impossible to study without the RT information.

We used models under the SSM family to fit tree shrew decision behaviors on the trial level. SSMs predict the choice and RT distribution with a mathematically defined dynamic decision-making process controlled by cognitively meaningful parameters and offer testable hypotheses about the underlying mechanisms. Signal detection models have also been used to explain perceptual decision-making behaviors (Newsome et al., 1989), but they only predict the choices made by subjects in a decision process, ignoring the information contained in the response time. Furthermore, the choice data are usually averaged over trials, further reducing the information present in the raw data. By comparison, SSMs have the advantage of maximizing the efficiency of the animal experiments and data analysis (Ratcliff et al., 2003).

Despite the RDM showing a slightly better Bayes factor than the TRDM in the exponential-delay group because of simplicity, the TRDM had the same ability to reproduce the observed choice and RT pattern. Together with its overwhelmingly better performance in the fixed-delay group, the TRDM was overall the better model for this dataset. By examining the source of the simulated data (Extended Data Fig. 4-1), we found that timer-induced random choices largely contribute to the plateau of a nonperfect accuracy in the easiest conditions. Canonically, this nonperfect accuracy is modeled by “lapse rate” under the Signal Detection framework (Wichmann and Hill, 2001; International Brain Laboratory et al., 2021; Wang et al., 2020; Prins, 2012). The lapses are usually assumed to happen via a Bernoulli process, i.e., the animals simply make guesses at some random rate independently from trial to trial, while providing no detailed process of choice generation. In comparison, the TRDM utilizes a time accumulator that is highly similar to evidence accumulation to generate random choices. It offers a more integrative solution to the interaction between evidence-based and stimulus independent mechanisms. This can be more plausible on the neuronal level than two separate processes that involve very different calculations. In addition, the TRDM provides the extra ability to explain why we rarely see extremely long RTs in the difficult conditions, especially in the equal-evidence conditions. The time accumulator can limit the RT so that the decision-makers do not waste too much time on a single decision when the evidence is obscure. Thus, we think that the TRDM has more explanatory power than models that include a “lapse rate.” Furthermore, a recent study showed that mice alternate between states, such as lapse or biased decisions, during a perceptual decision-making task, and they have a higher probability to stay in the same state for consecutive trials (Ashwood et al., 2022). Therefore, Bernoulli “lapses” would be an oversimplified explanation of how nonperfect choices happen. In future studies, the temporal sequence of choices and RTs should also be analyzed to further investigate the mechanism of decision state switching.

Finally, it is intriguing that the tree shrews in this study showed a fair amount of premature choices under fixed trial-delay although this strategy was suboptimal, in that it did not maximize the reward rate. The TRDM suggested that the animals actively applied a fast timer (or a short time limit) on the task without being trained to perform the task speedily. Interestingly, this tendency of rushing into choices was discouraged by the exponential trial-delay design that specifically punished fast incorrect responses more. The baseline suboptimal behavior could partly be because of (1) the characteristics of this animal model and/or (2) the stimulus design. The tree shrews showed much faster responses compared with humans on similar tasks (Kirkpatrick et al., 2021). They were very nimble and showed swift movements and reactions in various environments (behavior rig, home cage, nature, etc.). Given their motor capabilities, fast responses could be a survival strategy to guarantee the total amount of reward via high sampling frequency with slightly compromised accuracy, and could be broadly used in most scenarios to facilitate “exploration” behaviors, unless specifically discouraged. Additionally, in previous perceptual decision-making studies, stochastic stimuli with motion such as random dot kinematogram were usually used (Roitman and Shadlen, 2002; Ditterich, 2006; Resulaj et al., 2009). These stimuli require temporal integration to acquire evidence for choices. In our study, we used the static feature (contrast) as evidence. Although studies showed support for evidence accumulation even using the static stimuli in other species (Kirkpatrick et al., 2021), temporal integration might not be needed as strongly to generate a choice under this situation. This could result in short response times, leading the animals to a faster RT regime (more prone to make premature choices) and masking the effect of task difficulty on the RT (Fig. 2*G*, minor effect, although significant). Nevertheless, the tree shrew data emphasized the natural existence of f evidence-independent mechanisms in decision-making and offered an opportunity to examine their effects. These behavioral patterns also suggest that we should consider the involvement of processes in addition to the evidence accumulation process in other animal/human models when interpreting both behavioral and neural data from decision-making tasks. Here, we included an independent time accumulator to implement this additional process in our decision-making models (Hawkins and Heathcote, 2021). However, it should be noted that mechanisms other than the time accumulator could also generate the fast-guessing responses and our results do not rule out these possible mechanisms. In other words, the time accumulator was not necessarily the true underlying mechanism, but rather a piece of evidence for the involvement of multiple generative processes for decision instead of one single process. Other studies have indeed applied alternative approaches to account for decisions not entirely based on evidence accumulation, such as combining the decision process with a probabilistic fast-guess mode that generates a normally distributed guessing time (Ratcliff and Kang, 2021). Future studies that incorporate neural data will be needed to reveal exactly how response times in perceptual decision tasks are affected by information other than the sensory strength.

## Synthesis

Reviewing Editor: Nicholas J. Priebe, University of Texas at Austin Decisions are customarily a result of the Reviewing Editor and the peer reviewers coming together and discussing their recommendations until a consensus is reached. When revisions are invited, a fact-based synthesis statement explaining their decision and outlining what is needed to prepare a revision will be listed below. The following reviewer(s) agreed to reveal their identity: Santiago Jaramillo. Note: If this manuscript was transferred from JNeurosci and a decision was made to accept the manuscript without peer review, a brief statement to this effect will instead be what is listed below.

SYNTHESIS

Two reviewers evaluated the manuscript “Tree shrews as an animal model for studying perceptual decision-making reveal a critical role of stimulus-independent processes in guiding behavior” and both reviewers agreed that the revisions address the issues previously raised. The manuscript establishes a perceptual decision-making task using tree shrews, and demonstrates that, as with other mammals, tree shrew task training and behavior is influenced by stimulus-independent processes. The paper will be useful for future studies examining decision making in tree shrews. The reviewers did, however, have some minor concerns and questions. These issues are detailed below, which you should find useful in revising your manuscript.

Issues:

1) The authors agree that they cannot distinguish the TRDM from many other models that incorporate fast guessing. This is an extremely important caveat that should be clear to the reader. We appreciate that the authors emphasize stimulus-independent processes instead of the specific timing mechanism in the text and the additions to the last paragraph of the discussion. The authors should plainly state in the discussion that there is nothing in their results that specifically supports the time accumulator as the mechanism for fast guessing and that many other models that do not include a time accumulator might also explain the data.

2) “Even if we add an additional random guess mechanism into Snapshot models, the random guess probability will be independent of the stimulus strength, still unable to reproduce the relationship between the incorrect RTs and contrast difference observed in our data” (line 410). It is not clear that this statement is correct. In this model, the large majority of error trials in the easiest conditions would be generated by fast guesses, whereas error trials in the difficult conditions would be a mixture of fast guesses and snapshots (which have RTs longer than the fast guesses). Therefore, error RTs will follow the observed pattern. Further, using the authors’ logic, they should rule-out sequential sampling models in the fixed-delay data because they do not observe an effect of stimulus strength on RT. In more general terms, if the authors want to claim that the snapshot model with fast guessing cannot explain their data, then they should fit it to their data and compare it to the TRDM. This comparison is not necessary to support the claims outlined above, but it is critical that readers understand that the space of models that might explain the data is large.

3) It may not be helpful to characterize collapsing bounds or urgency signals as evidence-independent processes (they are mathematically equivalent). The bound is applied directly to the accumulated evidence. It also produces error RTs that are longer than correct RTs, which is the opposite of what the authors observed. Finally, collapsing bounds are the optimal solution in terms of maximizing reward rate when there is a mixture of stimulus difficulties (Drugowitsch et al, 2012). The useful distinction is between models with a single generative process for choice/RT and models that contain multiple.

4) Lines 390-396: It is difficult to understand the point the authors are conveying.

5) Many of the plots do not have ticks on the abscissa.

6) The authors may want to rephrase the sentence in the abstract that states their main results: “Specifically, they made occasional fast guesses under a constant increase in the delay between trials for incorrect responses that did not vary with the response time.” The current version is difficult to interpret because it is not clear what the last clause (“did not vary with response time”) refers to. Please consider splitting the sentence.

7) The units in Fig.1D are not correct. The vertical axis should be “fraction” (instead of %) or the values should be 0-100 instead of 0-1.

8) When presenting p-values (e.g., in line 276) the authors should indicate what statistical test was used.

The reviewers also included ideas that may be helpful, but that the authors are free to ignore.

1) A useful discussion point could be to consider why there was such a minimal effect of stimulus strength on RT. It is a major discrepancy between monkey and human data and a primary aim of the paper is to establish tree shrews as an alternative animal model for decision making. Is it a species-specific difference? Is it a training effect? Is it because of the specific stimulus used? Most studies in humans and monkeys with large modulation of RTs used a stochastic stimulus which yields noisy samples of evidence, which makes accumulating evidence more beneficial. The current study used a static stimulus. The authors also might find it useful to know (if they didn’t already) that it takes several months of training-after learning the basics of the task-for monkeys to show stimulus-dependent RTs.

2) It is surprising that the percentage of trials estimated to be induced by the timer wasn’t emphasized more. It suggests that 30-60% of trials in the fixed delay condition result from a stimulus independent generative process even though the psychometric functions look reasonably good. That’s a huge number! How does that estimate compare to the estimate yielded by fitting a lapse rate to the simulated choice data. If the estimate yielded by the lapse rate is lower than the true rate, it suggests that people might be underestimating just how corrupted their data is by stimulus-independent generative processes.

3) Is there a link between serial choice biases (e.g. win-stay, lose-switch) that are commonly observed and the fast guessing observed here. Do the animals exhibit these biases? An analysis that quantifies these biases separately for different RT quantiles could be interesting. Similarly, one could try to predict the animal’s RTs using the trial history. Most studies model serial biases as a weight on the decision process, but it could be that they result from a separate generative process which maps onto the fast guesses.
