# Supplementary material for: Tree Shrews as an Animal Model for Studying Perceptual Decision-Making Reveal a Critical Role of Stimulus-Independent Processes in Guiding Behavior
Source: eNeuro. 2022 Nov 24;9(6):ENEURO.0419-22.2022. doi: 10.1523/ENEURO.0419-22.2022 (PMC9718354; doi:10.1523/ENEURO.0419-22.2022)
Supplement: Extended Data Table 1-1 — Statistical table. Download Table 1-1, DOC file. [file enu-eN-NWR-0419-22-s06.doc]

**Table 1-1 Statistical Table**

| **Line** | **Data Structure** | **Type of Test** | **Confidence Interval** |
| --- | --- | --- | --- |
| a | Non-parametric | Mixed effect linear regression | (-.002, .019) |
| b | Non-parametric | Mixed effect linear regression | (-.103, -.046) |
| c | Non-parametric | Mixed effect linear regression | (-.034, -.009) |
| d | Non-parametric | Mixed effect linear regression | (-.084, -.009) |
