# Supplementary figures and images for: Tree Shrews as an Animal Model for Studying Perceptual Decision-Making Reveal a Critical Role of Stimulus-Independent Processes in Guiding Behavior
Source: eNeuro. 2022 Nov 24;9(6):ENEURO.0419-22.2022. doi: 10.1523/ENEURO.0419-22.2022 (PMC9718354; doi:10.1523/ENEURO.0419-22.2022)

**Table 1-2 TRDM Best Fitting Parameters of Each Animal**

| **Animal** | 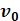 | 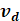 | 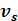 | 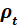 | 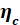 | 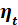 | 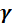 | 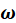 | 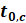 | **Log likelihood** |
| --- | --- | --- | --- | --- | --- | --- | --- | --- | --- | --- |
| 085 | 3.388 | 1.524 | 0.254 | 2.306 | 0.600 | 1.358 | 0.194 | 0.516 | 0.048 | 3541.048 |
| 087 | 2.825 | 2.532 | 0.809 | 4.672 | 0.753 | 1.486 | 0.343 | 0.562 | 0.049 | 5162.741 |
| 101 | 3.037 | 1.768 | 0.636 | 2.154 | 0.603 | 1.362 | 0.374 | 0.519 | 0.055 | 5363.755 |
| 123 | 2.383 | 1.556 | 0.162 | 0.100 | 0.703 | 1.702 | 0.459 | 0.501 | 0.047 | 943.074 |
| 125 | 2.253 | 3.933 | 0.948 | 3.394 | 1.074 | 0.422 | 0.978 | 0.573 | 0.110 | 7309.722 |
| 130 | 2.790 | 2.633 | 0.185 | 1.155 | 0.946 | 0.042 | 0.577 | 0.513 | 0.195 | 3070.600 |
| 131 | 2.856 | 3.476 | 0.806 | 1.485 | 1.161 | 0.053 | 0.611 | 0.505 | 0.125 | 3782.443 |
| 137 | 1.612 | 2.608 | 0.465 | 1.572 | 0.865 | 0.440 | 0.969 | 0.528 | 0.167 | 2445.965 |
| 138 | 2.327 | 4.142 | 0.979 | 1.155 | 1.294 | 0.043 | 0.223 | 0.518 | 0.151 | 3190.957 |

Supplement: Extended Data Table 1-2 — TRDM best fitting parameters of each animal. Download Table 1-2, DOC file. [file enu-eN-NWR-0419-22-s07.doc]

**Table 1-3 RDM Best Fitting Parameters of Each Animal**

| **Animal** | 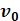 | 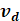 | 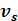 | 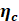 | 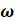 | 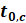 | **Log likelihood** |
| --- | --- | --- | --- | --- | --- | --- | --- |
| 085 | 3.122 | 1.243 | 0.161 | 0.904 | 0.498 | 0.000 | 2668.097 |
| 087 | 3.724 | 1.577 | 0.352 | 1.235 | 0.485 | 0.001 | 4494.682 |
| 101 | 2.877 | 1.436 | 0.329 | 0.865 | 0.540 | 0.000 | 4287.514 |
| 123 | 2.041 | 1.713 | 0.160 | 1.054 | 0.515 | 0.000 | 137.629 |
| 125 | 4.362 | 2.969 | 0.333 | 0.893 | 0.543 | 0.098 | 7251.003 |
| 130 | 2.730 | 2.602 | 0.228 | 0.924 | 0.511 | 0.192 | 3063.955 |
| 131 | 2.820 | 3.370 | 0.812 | 1.133 | 0.505 | 0.123 | 3773.603 |
| 137 | 2.114 | 2.196 | 0.330 | 0.759 | 0.523 | 0.149 | 2431.973 |
| 138 | 2.398 | 4.043 | 0.946 | 1.282 | 0.518 | 0.150 | 3183.084 |

Supplement: Extended Data Table 1-3 — RDM best fitting parameters of each animal. Download Table 1-3, DOC file. [file enu-eN-NWR-0419-22-s08.doc]

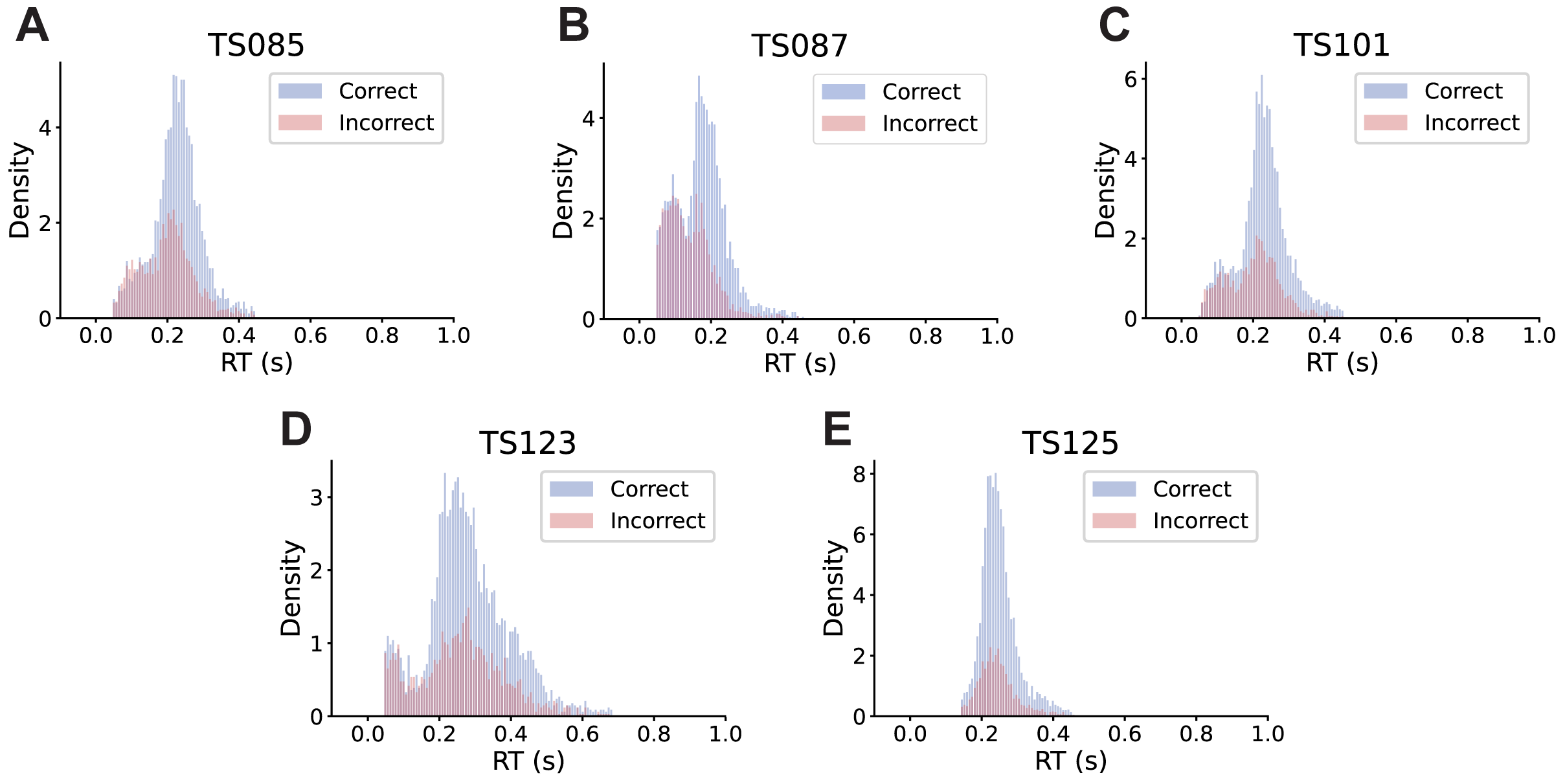

Supplement: Extended Data Figure 2-1 — Response time distributions of the individual animals from the fixed-delay group. Download Figure 2-1, TIF file. [file enu-eN-NWR-0419-22-s02.tif]

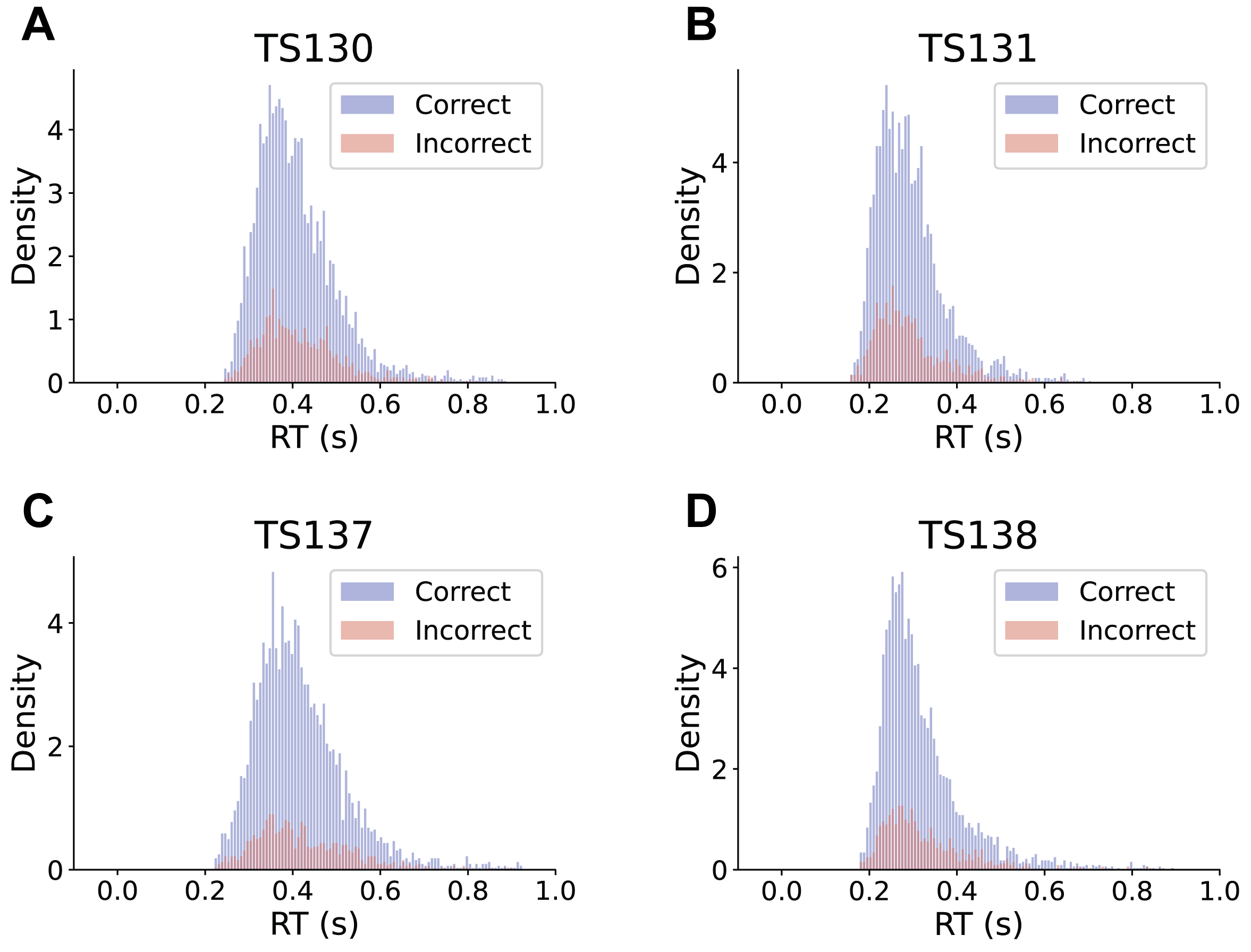

Supplement: Extended Data Figure 2-2 — Response time distributions of the individual animals from the exponential-delay group. Download Figure 2-2, TIF file. [file enu-eN-NWR-0419-22-s03.tif]

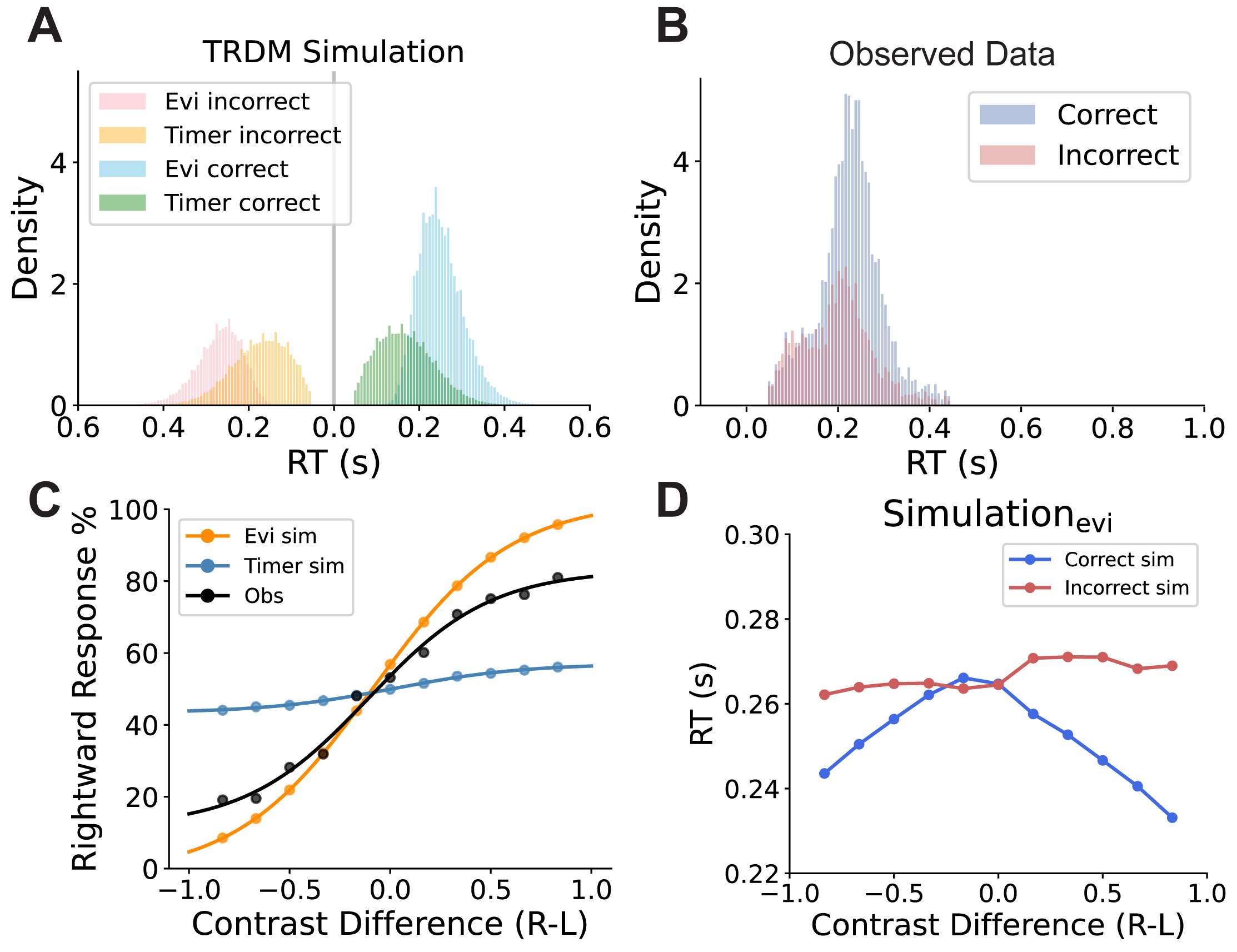

Supplement: Extended Data Figure 4-1 — Decomposition of an example animal’s simulated RT distribution by the TRDM. A, The simulated RTs for one example animal (TS085) from the first group are divided into four groups: evidence accumulator generated RT for correct (blue) and incorrect (pink) responses, and time accumulator generated RT for correct (green) and incorrect (yellow) choices. Compared with the observed data (B), the plots show that the TRDM interprets the first peak (fast RT) in the RT distribution as generated by the time accumulator. C, Simulated psychometric curves generated by the evidence accumulators and the time accumulator. D, Evidence accumulator simulated RT as a function of contrast difference. Download Figure 4-1, TIF file. [file enu-eN-NWR-0419-22-s04.tif]
